# VHrare study: Prevalence, clinical features and management of severe rare bleeding disorders in a large cohort

**DOI:** 10.1002/jha2.664

**Published:** 2023-03-09

**Authors:** Olga Benítez Hidalgo, Maria Fernanda Martinez Garcia, Irene Corrrales Insa, Mariana Fernández‐Caballero, Lorena Ramírez Orihuela, Vicente Cortina Giner, Natàlia Comes Fernández, Juan Carlos Juarez Gimenez

**Affiliations:** ^1^ Hematology Department Hospital Universitari Vall d'Hebron, Experimental Hematology, Vall d'Hebron Institute of Oncology (VHIO), Vall d'Hebron Barcelona Hospital Campus Barcelona Spain; ^2^ Medicine Department Universitat Autònoma de Barcelona Barcelona Spain; ^3^ Congenital Coagulopaties Banc de Sang i Teixits Barcelona Spain; ^4^ Transfusional Medicine, Vall d'Hebron Institut de Recerca Universitat Autònoma de Barcelona (VHIR‐UAB) Barcelona Spain; ^5^ Laboratory of Haematology, ICO‐Badalona, Germans Trias i Pujol University Hospital, Institut Josep Carreras Contra la Leucemia Universitat Autònoma de Barcelona Barcelona Spain; ^6^ Hematology Department Hemostasis Laboratory, Vall d´Hebron Hospital Barcelona Spain; ^7^ Pharmacy Department Hospital Universitari Vall d'Hebron Barcelona Spain

**Keywords:** bleeding phenotype, diagnosis, management, rare bleeding disorders

## Abstract

Introduction: Rare bleeding disorders (RBD) constitute 5% of total hereditary bleeding disorders, although the number could be higher, due to the presence of undiagnosed asymptomatic patients. The objective of this study was to analyze the prevalence and characteristics of patients with severe RBDs in our area.

Material and methods: We analyzed the patients with RBD followed at a tertiary‐level hospital between January 2014 and December 2021.

Results: A total of 101 patients were analyzed, with a median age at diagnosis of 27.67 years (range 0–89), of which 52.47% were male. The most frequent RBD in our population was FVII deficiency. Regarding the diagnostic reason, the most frequent cause was a preoperative test and only 14.8% reported bleeding symptoms at the time of diagnosis. A genetic study was carried out in 63.36% of patients and the most frequent mutation type found was finding a missense mutation.

Conclusions: The distribution of RBDs in our centre is similar to the one reported in the literature. The majority of RBDs were diagnosed from a preoperative test and this allowed preventive treatment prior to invasive procedures to avoid bleeding complications. 83% of patients did not have a pathological bleeding phenotype according to ISTH‐BAT

## 1 INTRODUCTION

Rare bleeding disorders (RBDs) constitute 2–5% of hereditary bleeding disorders in the general population and include deficiencies of factors I (fibrinogen), II, V, VII, X, XI and XIII. The remaining 95% are mostly due to deficiencies of factor VIII (haemophilia A), factor IX (haemophilia B) and von Willebrand factor (von Willebrand´s disease) [1].

The prevalence of RBDs in the general population is estimated to be 1 symptomatic case per 500,000 inhabitants, although the total number of patients could be higher due to the presence of undiagnosed asymptomatic patients and it may vary by country or region [1, 2]. Among RBDs, FVII deficiency is the most frequently diagnosed RBDs with an observed incidence between 1:300,000 to 1:500,000, which represent 30–50% of the total according to published series [4, 5, 6, 7, 8, 9, 10, 11], followed by FXI deficiency, which accounts for 23–39% according to registries [5, 8, 9]. The rare RBDs are FII and FXIII deficiencies with a prevalence of approximately 1 per 2 million inhabitants each [3, 8, 9], and combined factor deficiencies or congenital deficiency of vitamin K‐dependent factors, of which there are fewer than 30 cases reported worldwide until 2008 [12].

Symptoms of RBDs may vary depending on the type of deficiency and patient. These symptoms are usually related to the type of mutation causing the disease and the plasma levels of the deficient factor, which may cause more or less bleeding. [2]. Usually, with a factor level >20%, haemostasis is ensured to lead a normal daily life, though it should be individualised according to the factor deficiency and patient´s characteristics.

The diagnosis is initiated in patients with haemorrhagic symptoms and/or abnormal results in basic coagulation tests including activated partial thromboplastin time (aPTT), prothrombin time (PT), and thrombin time (TT). [13]. If abnormal results are found in these tests, a mixing test (50:50) is performed to rule out the presence of an inhibitor. When the mixing test corrects the coagulation tests, levels of clotting factors are determined. Although some hereditary bleeding disorders can be easily diagnosed through clinical evaluation and laboratory studies, genetic studies allow a more accurate and complete diagnosis for some of these conditions. [14] Nowadays, with Next Generation Sequencing techniques, different gene panels have been developed to approach the genetic study of all bleeding disorders with one common strategy which is faster, easier and cheaper than the individual study of each gene using traditional Sanger sequencing technology. [15]

The objective of this study was to analyze the prevalence and characteristics of patients with RBDs, with levels < 20% followed at a tertiary‐level hospital and to investigate whether there are correlations between factor levels and bleeding phenotype, as well as with the genetic mutation responsible for the disorder if available. The secondary endpoint was to compare the data reported in the literature.

## 2 MATERIALS AND METHODS

Due to the variability between the bleeding phenotype and factor levels, we conducted an observational retrospective study where we collected data from patients with RBDs followed at a tertiary‐level hospital.

A complete review was made of the medical charts of the patients diagnosed with RBDs with factor levels < 20%, less than 1 g/dl in case of fibrinogen deficiency, who attended a reference centre between January 2014 and December 2021. The cut‐off levels that we considered to classify patients as affected with a severe deficiency were < 20% following the CSUR (Reference Centres, Departments and Units) criteria.

Patients with factor levels > 20% were excluded, as well as patients with fibrinogen levels > 1 g/dl and those whose bleeding disorders were caused by anticoagulant therapy or other acquired bleeding disorders.

The variables studied were:

- Sex
- Age at diagnosis
- RBD type
- Baseline factor levels by one‐stage clotting assay
- Bleeding assessment by the ISTH Bleeding Assessment Tool (ISTH‐BAT)
- Genetic study if available
- Diagnostic reason
- Family history of RBD
- Haemostatic treatment

**What is the NEW aspect of your work?**

This study is the first study in which clinical data from south of Europe RBD patients is described and shows the highest rate of genetic testing compared with what was published until now.

**What is the CENTRAL finding of your work?**

The distribution of RBDs in our centre is similar to the one reported in the literature and It is striking that most patients with pathological bleeding scores are women.

**What is (or could be) the SPECIFIC clinical relevance of your work?**

The majority of RBDs were diagnosed from a preoperative test and this allowed prophylactic treatment prior to invasive procedures to avoid bleeding complications. This  shows the importance of preoperative coagulation tests and good anamnesis.

### 2.1 Clotting assays

In all cases, the one‐stage clotting assay was used in order to determine factor levels and for fibrinogen, prothrombin time‐derived fibrinogen tests and Clauss assay were performed. Samples were collected into tubes containing sodium citrate.

### 2.2 Bleeding phenotype

The bleeding phenotype was assessed by the ISTH‐BAT. The cut‐off value for an abnormal ISTH‐BAT score was ≥4 in adult men, ≥6 in adult women, and ≥3 in children [16]. The score was performed using data collected from the medical charts.

### 2.3 DNA extraction and sequencing

Samples of peripheral blood were collected into tubes containing citrate dextrose solution or ethylenediaminetetraacetic acid. Genomic DNA was isolated from 300 μl of blood using a QIAsymphony SP instrument and the QIAsymphony DNA Midi Kit (Qiagen), following the manufacturer´s protocol. The genetic study was afforded by a gene panel designed for the amplification of genes related to hereditary bleeding disorders. The protocol targeted all exons, intronic flanking regions and 5′UTR regions of the defined genes which were amplified by multiplex polymerase chain reactions. Library preparation, including patient‐specific indexation, was performed and libraries were pooled and pair‐end sequenced simultaneously using Reagent Kit v2 (300 cycles) in a MiSeq platform (Illumina).

### 2.4 Data analysis and mutation validation

Barcoded sequences were de‐multiplexed and analyzed individually. After variant calling, the resulting files were used as input for VariantStudio 2.2.1 (Illumina). The result of this step is the identification of potentially pathogenic variants and filtering of the polymorphisms described to date. Sanger sequencing was used to validate all putative mutations and to supplement all the exons/regions of the gene related to the clinical diagnosis displaying less than 10X read coverage. [17]

## 3 RESULTS

In total, 101 patients followed in our centre between January 2014 and December 2021 had severe RBDs based on our definition, with factor deficiency levels < 20% or fibrinogen < 1 g/dl. The minimum follow‐up time was 6 months. The overall characteristics of the series are summarized in Table 1. The most prevalent deficiency is FVII deficiency (36.63%). Data on the reason why the study was performed were collected being an abnormal basic coagulation test in a preoperative period the most common diagnostic reason (37.62%).

**TABLE 1:** Overall characteristics of the series°

	*n* = 101
Median age in years (range)	27.67 (0–89)
Male	53 (52.47%)
Type of deficiency	
*Fibrinogen*	15 (14.85%)
*FII*	0
*FV*	7 (6.93%)
*FVII*	37 (36.63%)
*FX*	4 (3.96%)
*FXI*	26 (24.74%)
*FXIII*	3 (2.97%)
*Combined deficiencies*	9 (8.91%)
– FV‐FVIII	– 8 (7.92%)
– FVII‐FXI	– 1 (0.99%)
Levels (%)	
≤*1*	33 (32.67%)
*2–10*	38 (37.62%)
*11–20*	30 (29.7%)
Genetic study available	64 (63.36%)
Diagnostic reason	
*Family study*	19 (18.81%)
*Bleeding symptoms*	15 (14.85%)
*Abnormal routine coagulation test*	16 (15.84%)
*Abnormal preoperative coagulation test*	38 (37.62%)
*Abnormal coagulation test during hospitalisation*	2 (1.98%)
*Unknown*	11 (10.89%)
History of spontaneous bleeding	22 (21.78%)
Need for replacement therapy	64 (63.36%)
Thrombotic events related to treatment	0

Only 18 patients (17.82%) reported abnormal bleeding at the time of diagnosis (regardless of the diagnostic reasons) and 48 (47.52%) reported it in their lifetime. Twenty‐ three per cent of our population had a pathological ISTH‐BAT score, and of these, 70% were female patients.

In contrast, 64 patients (63.36%) received replacement therapy in their lifetime due to haemorrhage or as prophylaxis before invasive procedures, the latter being the reason in 82% of patients.

Most patients (63.36%) had undergone a genetic study to find the mutation responsible for the deficiency. From those studies, 46% of the patients had either a homozygous mutation or 2 or more heterozygous mutations in the same gene. The most common mutation type in our severe patients was a missense mutation (70%).

The characteristics of the patients with each specific deficiency are summarised in Table 2 and their treatment is in Table 3.

**TABLE 2:** Patients’ characteristics with each specific deficiency

**Type of deficiency**	**Sex (%)**	**Age at Dx (years) median**	**Levels (%) mean (**± **SD)**	**ISTH‐BAT (median)**	**Genetic study**	**Replacement therapy**
**Fibrinogen (*n* = 15)**	M: 60	23 (0–36)	0.7 g/L (±0.2)	0 (0–7)	A: 93.3% NA: 6.6%	Episodic treatment: 53% Prophylaxis treatment: 6.6% No treatment: 40%
**FV (*n* = 7)**	M: 42.85	25.5 (0–74)	6.2 (±5)	0 (0–17)	A: 71.4% NA: 28.6%	Episodic treatment: 42.85% Prophylaxis treatment: 0% No treatment: 57.15%
**FVII (*n* = 37)**	M: 54	27 (0–63)	10.1 (±6.8)	0 (0–16)	A: 59.45% NA: 40.55%	Episodic treatment: 59.45% Prophylaxis treatment: 5.4% No treatment: 35.15%
**FX (*n* = 4)**	M: 50	1 (1–65)	10.9 (±6.74)	3 (0–15)	A: 0% NA: 100%	Episodic treatment: 0% Prophylaxis treatment: 50% No treatment: 50%
**FXI (*n* = 26)**	M: 53.84	45 (4–89)	5.7 (±4.9)	0 (0–10)	A: 61.53% NA: 38.47%	Episodic treatment: 50% Prophylaxis treatment: 0% No treatment: 50%
**FXIII (*n* = 3)**	M: 100	3 (1–4)	4.5 (±3.94)	7 (3–8)	A: 33% NA: 66%	Episodic treatment: 0% Prophylaxis treatment: 100% No treatment: 0%
**combined deficiencies (*n* = 9)**	M: 44.4	16.5 (5–59)	13.55 (±4.1)/26.25 (±11.89)	2 (0–12)	A: 33% NA: 66%	Episodic treatment: 66% Prophylaxis treatment: 0% No treatment: 33%

Abbreviations: A, available; Dx, diagnosis; M, male; NA, not available.

**TABLE 3:** Treatments in rare bleeding disorders

**Drug**	**Indication in RDB**	**Dosage**	**Available concentrated**
Fibrinogen	Congenital hypofibrinogaenemia and dysfibrinogaenemia	70 mg/kg	Riastap® 1 g^1^ Fibryga® 1 g ^2^
rFVIIa	FVII deficiency	15–30 mcg /4–6 h	Novoseven® 1, 2, 5^3^ and 8 mg
FXI	FXI deficiency	30–40 IU/kg	Factor XI‐Hemoleven®^4^
FX	FX deficiency	20–40 IU/kg	Factor IX/X Behring 1200 UI
FXIII	FXIII deficiency	Plasmatic FXIII: 40 IU/kg Recombinant FXIII: 35 IU/kg	Plasmatic FXIII: Cluvot®^6^ Recombinant FXIII: catridecacog, Novotherteen 2500UI®^7^

Abbreviations: FX, coagulation Factor X; FXI, coagulation Factor XI; FXIII, coagulation Factor XIII; rFVIIa, coagulation recombinant Factor VII activated.

^1^ ‐Riastap® *Ficha técnica*. [Consulted: august 2022] Available at: https://cima.aemps.es/cima/pdfs/es/ft/72725/72725_ft.pdf.

^2^ ‐Fibryga® *Ficha técnica*. [Consulted: august 2022] Available at: https://cima.aemps.es/cima/pdfs/es/ft/85322/FT_85322.pdf.

^3^ ‐Novoseven 5 mg ® *Ficha técnica*. [Consulted: august 2022] Available at: https://cima.aemps.es/cima/pdfs/es/ft/196006010/FT_196006010.pdf.

^4^ ‐Bauduer F, de Raucourt E, Boyer‐Neumann C et al. Factor XI replacement for inherited factor XI deficiency in routine clinical practice: results of the HEMOLEVEN prospective 3‐year postmarketing study. Haemophilia. 2015 Jul; 21(4):481‐9. doi: 10.1111/hae.12655. Epub 2015 Mar 26. PMID: 25817556; PMCID: PMC4657494.

^5^ ‐Factores IX/X Behering® 1200 UI. *Ficha técnica*. [Consulted: august 2022] Available at: https://cima.aemps.es/cima/pdfs/es/ft/65405/65405_ft.pdf.

^6^ ‐Cluvot® *Ficha técnica*. [Consulted: august 2022] Available at: https://cima.aemps.es/cima/pdfs/es/ft/78779/78779_ft.pdf.

## 4 DISCUSSION

This study is the first study in which clinical data from South of Europe RBD patients are described. The results of our series on the frequency of each type of factor deficiency are similar to those previously reported in other areas, except for the frequency of FX deficiency which is lower in our series. [1] In addition, our series show the highest rate of genetic testing compared with what is published now. Different reviews have been published in which a correlation has been established between the levels considered haemostatic in the different RBDs, which differentiates the risk of bleeding according to the factor level [9]. However, in our study, we established a cut‐off point of 20% factor levels, which is established for severe deficiencies according to the criteria requested for certification of CSUR of the National Health System in Spain. An exception to the levels considered haemostatic was the case of fibrinogen deficiency, where levels ≥ 1 g/dl were considered hemostatic. According to the literature, symptoms are more common in afibrinogenaemia cases and manifest as umbilical cord bleeding, delayed wound healing, and central nervous system (CNS) bleeding [18, 19, 20].

Cases with hypofibrinogenaemia and dysfibrinogenaemia are usually asymptomatic as they have fibrinogen levels of around 1 g/L, but they may have pathological bleeding secondary to trauma or surgery and in cases of dysfibrinogenemia, thrombotic events can also occur [19]. In this regard, patients in our series with levels below 0.8–1 g/dl did not experience spontaneous bleeding or postoperative complications as they received replacement therapy with fibrinogen concentrate prior to invasive procedures with no thrombotic events reported. Every mutation studied was missense and the most found was a substitution in exon 8 of the FGG gene (66.67%), although not all patients were related. As recommended in the literature, in our series treatment and prophylaxis were performed with fibrinogen concentrates. In no case was necessary to use fresh frozen plasma (FFP) or cryoprecipitate [13, 18].

FII was the most uncommon RBD as reported in the literature, therefore we did not find any patient with levels < 20% in our series [1, 3].

Regarding FV deficiency, severe bleeding symptoms occur in patients with FV levels < 5%, while patients with FV between 5%–20% have usually only mild symptoms [21]. Thus, none of the patients with levels between 10–15% in this series presented any spontaneous bleeding, and only one of them required FFP before surgery. According to the literature, in our series, the highest ISTH‐BAT score was observed on two female patients with levels < 1% who reported musculoskeletal and intracranial bleeding. It is striking that all patients who underwent the genetic study showed at least 2 heterozygous mutations involving more than one exon in the F5 gene. More than 60 reported mutations were found in the literature, although it is true that no complete deletions of the gene were found, so this is thought to be incompatible with life [21]. Since there is no FV concentrate available, treatment was based on administering FFP. Huang et al. have reported that in the event of serious bleeding, platelet concentrates may be administered, since they are an FV reservoir, and in refractory cases, recombinant activated factor VII (rFVIIa) may be considered as a bypassing agent, when necessary. [21] In our series, platelet transfusion was required in one patient due to a bleeding complication after brain surgery despite FFP transfusions.

In our series, FVII deficiency was the most common disorder as reported in the literature. The literature describes the bleeding phenotype as highly variable, patients usually have bleeding symptoms with levels < 20% [1]. However, in our series only 46% of patients with levels < 20% had experienced some type of bleeding, with only 29% of spontaneous bleeds. In this regard, two patients with levels below 1% have an ISTH‐BAT of 0. In this series, the most commonly reported bleeding was mucocutaneous bleeds, mainly epistaxis and heavy menstrual bleeding (HMB) in women and post‐traumatic/surgical bleeding as reported in the literature. [22] Sevenet et al. not recommended long‐term prophylaxis in FVII deficiency except in patients with a previous episode of severe bleeding. [23] Three of our patients with a hemorrhagic phenotype required regular long‐term prophylaxis with rFVIIa. Preoperative prophylaxis with rFVIIa was received by 72% of patients to prevent bleeding. No thrombotic events were reported.

Severe FX deficiency has been one of the most uncommon disorders in our series a somewhat lower incidence than reported in the literature [1]. Usually, patients with levels between 10% and 40% experience mild mucocutaneous bleeding symptoms, like our patients with levels of 20%, who never required replacement therapy. [24] Auerswald recommended continued prophylaxis only in patients with FX < 1% [25]. However, in our series, two patients with levels > 1% (8.4% and 4% respectively) received regular prophylaxis due to frequent musculoskeletal haemorrhages and HMB.

FXI deficiency is characterized because FXI levels correlate very poorly with the bleeding phenotype. The literature shows that bleeding usually occurs after trauma or surgery in areas with high fibrinolytic activity such as the oral and nasal cavity, and urinary tract, and in the case of women, HMB and partum‐related haemorrhages [26]. In our series, only 35% of patients had experienced bleeding symptoms, mainly after surgery, although one patient also reported HMB. On the other hand, 56.5% received prophylaxis before surgery, half with FFP and half with FXI. Although cases of thrombosis have been reported during FXI replacement, none of our patients experienced this complication [27].

Regarding the genetic study, patients had different types of mutations, as described in the literature when dealing with a population other than Ashkenazi Jews. However, most of our patients with an available genetic study had either a homozygous mutation or 2 or more heterozygous mutations, as would be expected in this type of rare bleeding disorder due to their autosomal recessive inheritance or variable penetrance [28]. Eighty‐one per cent of the mutations found were missense type.

According to the latest studies on FXIII deficiency, patients with levels < 10% will generally present bleeding symptoms. Patients with < 1% have a high risk of spontaneous and early bleeding such as umbilical cord and CNS bleeding, and also delayed cicatrization [29]. In our series, three patients with FXIII deficiency were diagnosed due to bleeding symptoms and their levels ranged from 0.9‐3.8%. The treatment of choice is plasma‐derived FXIII concentrate. However, two of our patients received recombinant FXIII in a clinical trial so they currently continue with the same prophylaxis every 4 weeks [30].

Finally, combined deficiencies of clotting factors have been reported, all with a very low prevalence. In our series, we found nine cases of combined FV‐FVIII deficiency and one patient with FVII and FXI deficiency. Patients with combined FV‐FVIII deficiency usually have levels ranging from 5% to 20%, so they usually do not manifest bleeding symptoms except after surgery or trauma. [31] In our series, 80% of patients had a predominance of FV deficiency over FVIII, only one case had lower FVIII than FV levels and presented the highest ISTH‐BAT score in this group. Regarding the genetic study, it was available in only two unrelated patients, and both had homozygous mutations in the *LMAN1* but in different exons/introns. FV‐FVIII deficiency patients are usually treated on demand. Replacement of FV can be achieved only through FFP or platelet transfusion and for FVIII replacement, a large number of products are available, including FFP and FVIII concentrates. [31]. In our series, all but one patient has required prophylaxis prior to surgery with desmopressin, FVIII concentrates, or FFP.

One patient had combined FVII and FXI deficiency, with levels of 19% and 41%, respectively. The only symptom reported was epistaxis and no haemostatic treatment was required. This type of combined factor deficiency is very rare, and we have only found one family with the same type of deficiency in the literature [32]. Our patient presented genetic mutations in *F7* and *F11* separately, differentiating this combined deficiency from the FV‐FVIII where patients usually present with a common genetic mutation in one gene such as the *LMAN1* or *MCFD2* gene. [33]

Twenty‐three per cent of our population had a pathological ISTH‐BAT score, and of these, 70% were female patients, which suggests that women are more prone to bleeding, so it is particularly important to make an appropriate diagnosis and follow‐up in these patients. Joline L. Saes, et al recently published their real‐life data from the RBiN study showing, among other data, that over 70% of women with RBD report HMB or another gynaecological bleeding which might increase the ISTH‐BAT score in these patients. [34]

In conclusion, the distribution of RBDs in our area is similar to the one reported in the literature. Most RBDs were diagnosed based on an abnormal preoperative coagulation test, and this allowed for haemostatic preparation before surgery in 37.62% of patients, thus avoiding perioperative complications. Most of these patients (83%), despite having levels classified as severe, do not have a pathological bleeding phenotype according to the ISTH‐BAT score, and the ones who do have pathological scores have usually the lowest factor levels. It is striking that most patients with pathological scores are women (70%). Regarding the correlation between the genetic mutation and the bleeding phenotype, it was observed that among patients with genetic studies available, the most common mutation type was a missense mutation (70%) and that 46% of the patients with genetic studies had either a homozygous mutation or two or more heterozygous mutations in the same gene, which were usually the patients with lower factor levels.

## CONFLICT OF INTEREST

The authors declare no conflict of interest.

## Data Availability

N/A
